# Development of New Tuberculosis Vaccines: A Global Perspective on Regulatory Issues

**DOI:** 10.1371/journal.pmed.0040252

**Published:** 2007-08-07

**Authors:** Michael J Brennan, Uli Fruth, Julie Milstien, Rosemary Tiernan, Sergio de Andrade Nishioka, Liliana Chocarro, Developing Countries Vaccine Regulatory Network and the Ad Hoc Regulatory and TB Expert Panel

## Abstract

What are the regulatory challenges for testing and introducing investigative TB vaccines into countries where the disease is endemic?

In May 2005, the TB Vaccine Initiative of the World Health Organization (WHO) Initiative for Vaccine Research convened a working meeting of regulators, investigators, and clinicians from developing and developed countries involved in tuberculosis (TB) vaccine regulation and research (see Text S1 for a list of participants). The purpose of the meeting was to specifically discuss the regulatory challenges for testing and introducing investigative TB vaccines into countries where the disease is endemic.

A particular focus of this meeting was a discussion among representatives of regulatory authorities from the Developing Countries Vaccine Regulators Network (DCVRN) with those of the United Kingdom and the United States about the important challenges that each regulatory agency will need to address if effective new TB vaccines are to be registered in their countries. The DCVRN is a new WHO initiative establishing a network of vaccine regulators from nine countries: Brazil, China, Cuba, the Republic of South Korea, India, Indonesia, the Russian Federation, South Africa, and Thailand. It provides a forum for discussion, advancement of knowledge, and exposure to policies and procedures pertaining to evaluation of clinical trial proposals and clinical trial data.

In this article we describe key vaccine and regulatory issues arising during the meeting. We propose innovative recommendations that may be used to make important decisions for proceeding into various stages of clinical trials and for the final registration of new TB vaccines. We hope that this article will be particularly valuable to regulatory authorities of developing countries.

**Summary Points**

- Regulatory authorities from developing countries should be actively engaged in both product and clinical review of TB vaccines being investigated within their nation.
- In order to generate data that can be extrapolated to countries with genetically and environmentally diverse populations, it is important to perform multi-center clinical trials of new TB vaccines.
- Global mechanisms are needed for sharing information among regulatory authorities from countries engaged in the production and clinical testing of TB vaccines.
- There is no clear global regulatory pathway for TB vaccines.

## TB Vaccine Development: The Regulatory Challenges

As new vaccines are developed for TB and other infectious diseases that have an enormous impact on developing countries, clarification of the regulatory issues surrounding the development and possible licensure of these vaccines is becoming increasingly important.

In countries where national regulatory authorities (NRAs) are robust and have experience in the steps leading to marketing authorization for innovative products, there may not be an appropriate population in which to conduct clinical trials to establish the safety and efficacy of a new TB vaccine. Regulatory authorities in developed countries may lack sufficient experience with the disease burden of the target and other concomitant diseases. In addition, these regulatory agencies may be unfamiliar with medical treatment norms in countries with a high burden of TB. All these obstacles make it difficult for regulatory authorities in developed countries to address some of the regulatory and clinical development issues for TB vaccines. Conversely, in countries where TB is more prevalent, the regulatory agencies may be relatively inexperienced in the regulation of “first in human” products, or may lack the resources and regulatory framework to guide the development of a new TB vaccine product.

Given these difficulties, a partnership among regulatory authorities worldwide would be beneficial to the TB community, which faces many preclinical, clinical, and regulatory challenges that must be addressed during the development of effective new TB vaccines (Box 1).

**Box 1. Major Challenges for TB Vaccine Development**

- How can preclinical tests be better used to decide which new candidate TB vaccines will move forward into clinical testing?
- What is the best way to determine correlates of protection for TB vaccines?
- What partnerships can be developed to assure the ethical testing of new TB vaccines in developing countries?
- Will it be possible to replace the current BCG vaccine in developing countries?
- In populations with high rates of latent TB and HIV infection, can we safely perform clinical testing of new TB vaccines?
- Can TB be accurately diagnosed in pediatric populations?
- Is it possible to shorten treatment of TB by developing an effective immunotherapeutic TB vaccine?
- What global mechanisms can be developed to improve the ability of NRAs from developing countries to approve the clinical testing and registration of vaccines such as a new vaccine for tuberculosis?

## Introducing a New Generation of TB Vaccines into Developing Countries

An effective vaccine to both prevent TB and reduce its transmission is urgently needed because of the complexities surrounding the diagnosis, management, and treatment of TB, coinfection with TB and HIV, and challenges related to the treatment of drug-resistant Mycobacterium tuberculosis strains. Fortunately, several new TB vaccine candidates have been identified and a few have been formulated into vaccine products that are currently in phase I or II clinical trials [1,2]. A summary of first-generation TB vaccines currently in clinical trials and the next generation of TB candidate vaccines in various stages of development is provided in Table 1.

**Table 1:**
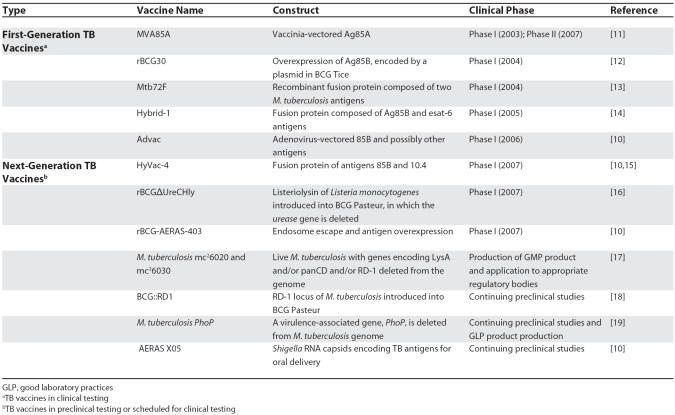
Summary of TB Vaccines Currently Under Development

Although it is generally acknowledged that a new safe and effective TB vaccine is needed, it may not be universally accepted, by all countries, that replacing the current bacille Calmette-Guérin (BCG) vaccine is in fact necessary or easily accomplished. However, with an integrated approach, for example using the current BCG vaccine in a prime–boost strategy, vaccination can make a major contribution to the elimination of TB [3,4].

Comprehensive preclinical testing of vaccine products is needed to provide supportive evidence for the safety, immunogenicity, and potential effectiveness of the vaccine as well as for estimating vaccine dose and immunization schedules for clinical testing in human populations. Preclinical and nonclinical tests for regulatory purposes include the development of critical product tests such as potency/stability assays, toxicology assays relevant to vaccines, and safety assays (see [5,6] and http://www.fda.gov/cber/index.html). A recent publication on the development of live TB vaccines [7] discusses useful methods for characterizing live TB vaccine products using good manufacturing practices (GMP). Also, Rowland et al. have described a number of tests that have been used to characterize the three major types of TB vaccine candidates: adjuvanted subunit vaccines, genetically modified vaccines, and vectored vaccines [8].

The evaluation of new TB vaccine candidates in clinical trials will depend on several factors, including: (1) characteristics of the target study population, (2) the incidence of TB within the population, and (3) the incidence of atypical mycobacteria and of other infectious diseases such as HIV and malaria in the region where the clinical trial is being conducted [3,6]. New preventive or therapeutic TB vaccines will need to be studied in several different clinical settings and in different populations, such as those who are “TB naïve” (prior to infection), those with latent TB infection (“sensitized”), and those who have TB disease. In developing countries, key issues will include informed consent and access to immunization by the target population, as well as other risk/benefit and ethical issues.

Currently no immunologic correlate or surrogate marker for protection against TB infection or TB disease has been defined. Therefore safety and immunogenicity studies will need to provide data that allow researchers to make a reasonable decision based on the best science for moving forward into the next stage of clinical vaccine development within the shortest time frame possible (see http://www.fda.gov/oc/gcp/). Feasibility studies of new TB vaccines will need to address: (1) the choice of comparative controls for evaluating safety and efficacy, (2) determination of vaccine dose, (3) assessment of vaccine-induced human immune responses, (4) enrollment eligibility, particularly for persons previously infected with M. tuberculosis, and (5) retention of clinical trial materials for future studies [9].

## Regulatory Strategies in TB Vaccine Development

To date there is no clear global regulatory pathway for new TB vaccine candidates; thus there is an urgent need to develop a “regulatory plan” prior to the start of phase III trials, which are just a few years away. Many of the regulatory issues to be addressed are in highly technical areas, such as characterization of the product using GMP, where the US Food and Drug Administration (FDA) and the European Agency for the Evaluation of Medicinal Products (EMEA) have vast experience. Experienced regulatory agencies, such as the FDA and EMEA, can contribute to the process by: (1) reviewing investigational new drug submissions for vaccines undergoing clinical trials (FDA) or (2) providing a scientific opinion for vaccines manufactured in Europe for use in developing countries but not in the European market (EMEA). In addition, the FDA and EMEA may work with individual national regulatory authorities or with networks of regulators in the regions, and/or provide experts to global or regional panels advising on vaccine safety and efficacy issues as well as on clinical trial protocols.

Other major regulatory issues are related to clinical study of the vaccines and include: (1) the standards of care used in clinical trials, (2) the review and monitoring of clinical trials by the regulatory authorities of the target countries, (3) the suitability of regional clinical data for registration purposes, and (4) the role of ethics committees or institutional review boards overseeing the trials. These critical regulatory issues need to be integrated into any new TB vaccine strategy. The development of a regulatory pathway is also critical for the vaccine developer or manufacturer, the sponsors of clinical trials, and health ministries in disease-endemic countries, since this pathway will have an impact on many decisions leading to the eventual introduction of a new TB vaccine.

Most regulatory authorities from developing countries need strengthening in the areas of preclinical product review and protocol review for initial phase I testing of new TB vaccines. While progress is being made through the DCVRN and regional initiatives, there is a continued need to develop the resource capacities of regulatory agencies within developing countries. Until this occurs, the most efficient regulatory path is to have the first TB vaccine phase I studies in TB-naïve study participants occur in countries nonendemic for TB with established regulatory authorities. It is likely that developing country regulatory authorities will then accept more advanced phase II and III clinical testing of TB vaccines in their regions.

Of critical importance are information sharing principles, which need to be established to foster interactions among all national regulatory authorities. Arguably, the sharing of product review experience among regulatory authorities from both developing and developed countries with their different perspectives and strengths would greatly facilitate vaccine introduction. Such sharing of experience would most likely require that both vaccine sponsors and regulatory organizations allow the sharing of proprietary product and clinical data, which may be done under product-specific agreements. A summary, from a developing country perspective, on regulatory actions that can be taken to accelerate the development of TB vaccines is shown in Box 2.

**Box 2. A Summary of Regulatory Challenges in TB Vaccine Development**

Currently it is easier for NRAs from developing countries to approve the clinical testing of new vaccines if product and early phase clinical testing have already been approved by a major regulatory authority in a developed country. The capacity to perform a thorough product and clinical testing review of an original vaccine application is a critical need of NRAs in developing countries.

It will also be beneficial to perform studies of the clinical TB vaccine lot in animal models of TB (particularly nonhuman primate models) that mimic the intended clinical application of a TB vaccine in human populations. Such animal studies would measure specific immune responses in hopes of identifying correlates of immunity. Preclinical studies that are used to meet regulatory requirements, including tests for safety and for an immune response that may be correlated with efficacy in humans, should use the same vaccine product that will be used in human clinical trials.

New TB vaccines should be compared with the BCG vaccine and TB diagnostics in current use within the developing country, since regulatory authorities may request comparisons with the products currently licensed in their country. A practical strategy is giving a new TB vaccine as a booster to adolescents that have been previously immunized with BCG vaccine. This approach may have fewer safety concerns compared with other immunization strategies and more defined case definitions and clinical endpoints, and could have the maximum impact on the transmission of TB. In a country highly endemic for HIV, it may be important to boost at a younger age before sexual activity and the risk of acquiring HIV. HIV-infected individuals should receive the boost before progression to AIDS.

BCG replacement clinical trials using live TB vaccines will be very difficult to approve within the current regulatory process of developing countries using BCG vaccine at birth. Use of new TB vaccines in pediatric populations will require thorough age-restricted de-escalation clinical studies (e.g., safety studies in adults and adolescents before initiating infant studies).

Clinical trial protocols should use the “standard of care” guidelines developed within the target country. Clinical investigations of new TB vaccines in developing countries need to provide tangible and sustainable outcomes for the population, such as continued trial site structure and the possibility of receiving a licensable TB vaccine in the future.

There may be a benefit for simultaneous submission of applications for clinical testing of a new TB vaccine to NRAs of developing countries (such as members of the DCVRN) to encourage cooperative review of the vaccine protocols among the various nations. This should improve the quality of the review, as well as shorten the time of approval.

Clinical practices that address the challenges outlined in Box 2 need to be established for the regulatory agencies involved in new candidate TB vaccine development. Such practices should be part of building the regulatory capacity in these countries while addressing the different risk/benefit perspectives that are appropriate for disease-endemic countries. Assessment of the need for TB vaccines in each region, particularly for the most relevant target populations, is critical for determining the approach each country will take in testing and regulating a new TB vaccine.

Three major immunization strategies are currently under consideration: (1) replacement of BCG with a more effective vaccine that provides longer lasting protection, (2) the introduction of a new vaccine into adolescents/adults that prevents adult pulmonary TB, and (3) reducing the treatment regimens for TB by use of an effective immunotherapeutic TB vaccine. Currently, a prime–boost strategy using a new TB vaccine candidate to boost the BCG vaccine that is routinely given in each country is considered the best way to test and introduce new TB vaccines into endemic countries that immunize with BCG vaccine at birth [4,10]. The regulatory authorities of each country willing to participate in human studies of new TB vaccine candidates will have a critical role in implementing these TB vaccine strategies. Such a role will include assuring product quality, monitoring clinical trials, and eventually licensing safe and effective new TB vaccines.

## Supporting Information

Text S1 List of Meeting Participants Found at doi:10.1371/journal.pmed.0040252.sd001
(95 KB PDF).

## Abbreviations

BCG: bacille Calmette-Guérin
DCVRN: Developing Countries Vaccine Regulators Network
EMEA: European Agency for the Evaluation of Medicinal Products
FDA: Food and Drug Administration
GMP: good manufacturing practices
NRA: national regulatory authority
TB: tuberculosis
WHO: World Health Organization
