# Supplementary material for: Development of New Tuberculosis Vaccines: A Global Perspective on Regulatory Issues
Source: PLoS Med. 2007 Aug 7;4(8):e252. doi: 10.1371/journal.pmed.0040252 (PMC1939860; doi:10.1371/journal.pmed.0040252)
Supplement: Supplementary file 1 [file pmed.0040252.sd001.pdf]

List of Meeting Participants:

DCVRN: **Dr Joey Gouws**, Inspectorate and Law Enforcement Cluster: Medicines Regulatory Affairs, Department of Health, Pretoria, South Africa; **Dr Yin Hongzhang,** State Food and Drug Administration, Beijing, People's Republic of China; **Dr Seog Youn Kang,** Department of Pharmacology, National Institute of Safety Research, Seoul, Republic of Korea; **Dr Yupin Lawanprasert,** Food and Drug Administration, Ministry of Public Health, Nonthaburi, Thailand; **Dr N.V. Medunitsin,** Tarassevich State Research Institute for Standardization and Control of Medical Biological Preparations, Moscow, Russian Federation; **Dr Sérgio de Andrade Nishioka**, Agência Nacional de Vigilância Sanitária, Brasilia, Brazil; **Dr Deybis Orta Hernandez,** Center for State Control of Drug Quality, Habana, Cuba; **Dr A.B. Ramteke,** Directorate General of Health Services, New Delhi, India; **Ms Antonia Retno Tyas Utami,** Sub-directorate of Copy Drug and Biological Product Evaluation, National Agency of Drug and Food Control, Jakarta, Indonesia; ***Regulatory and TB Vaccine Experts:*** **Dr Lew Barker,** Aeras Global TB Vaccine Foundation, Rockville, USA; **Dr Michael J. Brennan**, Food and Drug Administration - Center for Biologics, Evaluation & Research, Bethesda, USA; **Dr Daniel F. Hoft**, St. Louis University Health Sciences Center, St. Louis, USA; **Dr Tony Hawkridge,** Department of Paediatrics and Child Health, University of Cape Town, Rondebosch, South Africa; **Dr Harriet Mayanja-Kizza,** Makerere Medical School, Kampala, Uganda; **Dr Helen McShane,** Centre for Vaccinology and Tropical Medicine, Oxford, United Kingdom; **Dr Mair Powell**, Medicines and Healthcare Products, London, United Kingdom; **Dr Christine Sizemore,** National Institute for Allergy and Respiratory Diseases National Istitutes of Health, Bethesda, USA; **Dr**

Rosemary Tiernan, Food and Drug Administration-Center for Biologics Evaluation and Research, Rockville, USA; **Dr Barry Walker,** National Institute for Biological Standards and Control, Potters Bar, United Kingdom ***WHO secretariat:*** **Dr. Uli Fruth,** Initiative for Vaccine Research, Immunization, Vaccines and Biologicals; **Dr. Ivana Knezevic**, Quality, Safety and Standards; Dr. **Liliana Chocarro**, Quality, Safety and Standards, Immunization, Vaccines and Biologicals, World Health Organization, Geneva, Switzerland**.**
